# CP-673451, a Selective Platelet-Derived Growth Factor Receptor Tyrosine Kinase Inhibitor, Induces Apoptosis in *Opisthorchis viverrini*-Associated Cholangiocarcinoma via Nrf2 Suppression and Enhanced ROS

**DOI:** 10.3390/ph17010009

**Published:** 2023-12-20

**Authors:** Jinchutha Duangdara, Boonyakorn Boonsri, Apinya Sayinta, Kittiya Supradit, Pakpoom Thintharua, Supeecha Kumkate, Chinnawut Suriyonplengsaeng, Noppadol Larbcharoensub, Somkit Mingphruedhi, Narongsak Rungsakulkij, Paramin Muangkaew, Pongsatorn Tangtawee, Watoo Vassanasiri, Wikran Suragul, Tavan Janvilisri, Rutaiwan Tohtong, David O. Bates, Kanokpan Wongprasert

**Affiliations:** 1 Department of Anatomy, Faculty of Science, Mahidol University, Bangkok 10400, Thailand; jinchutha.dua@student.mahidol.ac.th (J.D.); boonyakorn.b@psu.ac.th (B.B.); kittiya.s@rumail.ru.ac.th (K.S.); pakpoom.thi@mahidol.ac.th (P.T.);; 2 Division of Health and Applied Sciences, Faculty of Science, Prince of Songkla University, Songkhla 90110, Thailand; 3 Division of Basic and Medical Sciences, Faculty of Allied Health Sciences, Pathumthani University, Pathum Thani 12000, Thailand; 4 Department of Radiological Technology, Faculty of Science, Ramkhamhaeng University, Bangkok 10240, Thailand; 5 Chakri Naruebodindra Medical Institute (CNMI), Faculty of Medicine Ramathibodi Hospital, Samut Prakan 10540, Thailand; 6 Department of Biology, Faculty of Science, Mahidol University, Bangkok 10400, Thailand; 7 Department of Pathology, Faculty of Medicine Ramathibodi Hospital, Mahidol University, Bangkok 10400, Thailand; noppadol.lar@mahidol.ac.th; 8 Department of Surgery, Hepato-Pancreato-Biliary Division, Faculty of Medicine Ramathibodi Hospital, Mahidol University, Bangkok 10400, Thailand; somkit.m@gmail.com (S.M.); watoo.vas@mahidol.ac.th (W.V.); wikran.sur@mahidol.ac.th (W.S.); 9 Graduate Program in Molecular Medicine, Faculty of Science, Mahidol University, Bangkok 10400, Thailand; 10 Department of Biochemistry, Faculty of Science, Mahidol University, Bangkok 10400, Thailand; rutaiwan.toh@mahidol.ac.th; 11 Centre for Cancer Sciences, Division of Cancer and Stem Cells, Biodiscovery Institute, University of Nottingham, Nottingham NG7 2RD, UK

**Keywords:** cholangiocarcinoma, *Opisthorchis viverrini*, CP-673451, PDGFR inhibitor, Nrf2

## Abstract

Platelet-derived growth factors (PDGFs) and PDGF receptors (PDGFRs) play essential roles in promoting cholangiocarcinoma (CCA) cell survival by mediating paracrine crosstalk between tumor and cancer-associated fibroblasts (CAFs), indicating the potential of PDGFR as a target for CCA treatment. Clinical trials evaluating PDGFR inhibitors for CCA treatment have shown limited efficacy. Furthermore, little is known about the role of PDGF/PDGFR expression and the mechanism underlying PDGFR inhibitors in CCA related to *Opisthorchis viverrini* (OV). Therefore, we examined the effect of PDGFR inhibitors in OV-related CCA cells and investigated the molecular mechanism involved. We found that the PDGF and PDGFR mRNAs were overexpressed in CCA tissues compared to resection margins. Notably, PDGFR-α showed high expression in CCA cells, while PDGFR-β was predominantly expressed in CAFs. The selective inhibitor CP-673451 induced CCA cell death by suppressing the PI3K/Akt/Nrf2 pathway, leading to a decreased expression of Nrf2-targeted antioxidant genes. Consequently, this led to an increase in ROS levels and the promotion of CCA apoptosis. CP-673451 is a promising PDGFR-targeted drug for CCA and supports the further clinical investigation of CP-673451 for CCA treatment, particularly in the context of OV-related cases.

## 1. Introduction

Cholangiocarcinoma (CCA) is a type of cancer that originates from cells in the bile ducts, specifically cholangiocytes or bile duct epithelial cells. It is a significant health concern, particularly in the northeast region of Thailand, where *Opisthorchis viverrini* (OV) is endemic. There is a link between liver fluke infection caused by OV, which is acquired through the tradition of consuming raw fish, and the development of CCA. OV migrates to the bile duct, leading to chronic inflammation and periductal fibrosis, ultimately contributing to CCA genesis [1,2]. CCA is characterized by a rich desmoplastic stroma, which contains a large number of cancer-associated fibroblasts (CAFs). These CAFs interact in reciprocal communication with tumor cells, contributing to the aggressiveness of CCA, cancer growth, progression, and drug resistance [3]. Due to the lack of effective early detection methods and treatments, CCA patients are often diagnosed at an advanced stage, resulting in high mortality rates [4]. Although the combination of gemcitabine and cisplatin is the standard first-line chemotherapy treatment for metastatic CCA, the median overall survival remains moderate at 19.5 months [5].

In recent years, a novel treatment approach known as “targeted therapy,” focusing on specific molecules of cancer cells, has shown highly effective antitumor effects in various solid tumors. Receptor tyrosine kinase (RTK), commonly upregulated in CCA, has been identified as a key mediator in promoting CCA progression, with a high RTK expression correlating with poor clinical outcomes [6,7]. Thus, targeting RTK in CCA treatment has shown promise for CCA treatments. Ongoing clinical trials are exploring drugs targeting various RTK subfamilies, including the fibroblast growth factor receptor (FGFR) [8], epidermal growth factor receptor (EGFR) [9], hepatocyte growth factor receptor (MET) subfamilies [10], vascular endothelial growth factor receptor (VEGFR), and platelet-derived growth factor receptor (PDGFR) [11].

PDGFR inhibitors have demonstrated effective antitumor activity in several solid tumors, including gastrointestinal stromal tumors, prostate cancer, non-small cell lung cancer (NSCLC), and CCA [11,12,13,14]. The PDGF family members and their receptors (PDGFR) play critical roles in tumor progression via crosstalk actions between cancer cells, CAFs, and stromal cells in the tumor microenvironment [15]. PDGFR comprises two isoforms, PDGFR-⍺ and PDGFR-β, belonging to the RTK family. The bindings of PDGFR with their cognate ligands, such as PDGF-A, PDGF-B, PDGF-C, and PDGF-D, induce receptor dimerization, autophosphorylation of the tyrosine residues, and activation of downstream signaling pathways, such as the phospholipase Cγ (PLC-γ), mitogen-activated protein kinase (Ras-MAPK), and phosphatidylinositol 3-kinase (PI3K) pathways [16]. These pathways stimulate multiple cellular responses, such as cell proliferation, motility, survival, and the recruitment of stromal cells, contributing to the tumor microenvironment [17].

There is evidence that the overexpression of PDGF-A and PDGFR-⍺ is linked to OV infection in CCA. This overexpression plays a critical role in the development and survival of CCA [18]. Studies on mouse models have demonstrated that PDGF and PDGFR are regulated between cholangiocytes and fibroblasts, contributing to biliary tract fibrogenesis and inflammation [19]. Therefore, PDGFR is a potential target for CCA therapy. While multitarget kinase inhibitors like imatinib and sunitinib have shown promise in disrupting PDGFR signaling, they can inhibit CCA cell proliferation, migration, and invasion [20] and sensitize CCA cells to TRAIL-induced apoptosis [21]. However, the clinical effectiveness of these agents in CCA has not yielded a highly successful outcome.

Recently, a powerful selective inhibitor of PDGFR kinase, CP-673451, has been developed, demonstrating more than a 450-fold inhibition specifically for PDGFR compared to other receptors [22]. CP-673451 effectively promotes apoptosis in NSCLC cells and exerts strong inhibition on cell migration. In a preclinical study, CP-673451 demonstrated the suppression of cancer growth and induction of apoptosis in an A549 xenograft nude mouse model without significant cytotoxic effects [23]. Thus, CP-673451 emerges as an attractive therapeutic agent for CCA. Moreover, previous studies have shown that PDGF and PDGFR play essential roles in cancer development and progression, including CCA [18]. Therefore, understanding the expression of PDGF and PDGFR in CCA patient tissues can provide valuable insights into the potential efficacy of CP-673451 as a targeted therapy for CCA. To determine whether targeting PDGFR holds potential as a therapeutic approach for CCA, our study examined the expression of PDGF and PDGFR in OV-related CCA patient tissues, as well as the anti-tumor effects of CP-673451.

## 2. Results

### 2.1. Expression Level of PDGFs and PDGFRs in CCA Tissues and CCA Cell Lines

**Figure 1:**
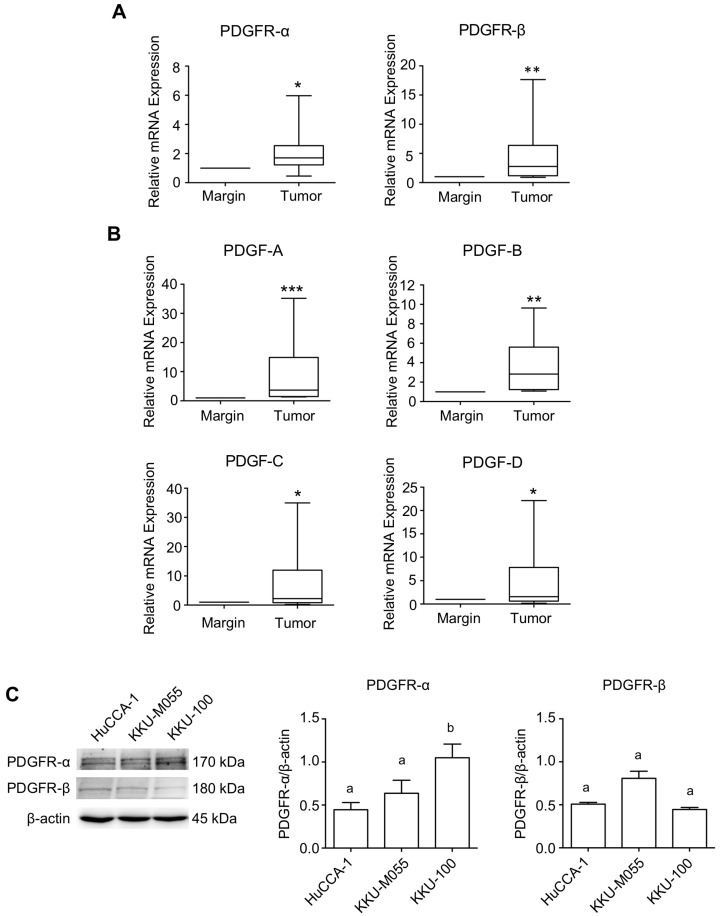
Expression level of PDGF-PDGFR in Thai-CCA tissues and OV-related CCA cell lines. mRNA expression of (**A**) PDGFR and (**B**) PDGF ligands in paired CCA and normal margin tissue, determined by RT-qPCR. Results are presented as box-and-whisker plots with a 5–95% confidence interval. Statistical comparisons were conducted using the Wilcoxon matched-pairs test. *, **, and *** indicate significant difference from pair normal margin tissues at *p* < 0.05, *p* < 0.01, and 0.001, respectively. (**C**) Analysis of basal PDGFR-α and PDGFR-β protein expressions in different CCA cell lines by Western blot analysis. The level of protein expression was normalized with the internal control, β-actin. Different letters indicate significant differences among cell lines in each protein marker, *p* < 0.05.

The mRNA analysis revealed high expression levels of PDGFR-α and PDGFR-β, along with their cognitive ligands, PDGF-A, PDGF-B, PDGF-C, and PDGF-D, in tumor tissue compared to tissue resection margins (Figure 1A,B). The relationships between the mRNA expression of tumoral PDGFR-α, PDGFR-β, and PDGF ligands are summarized in Table S1 (Table S1 in Supplementary Materials). Investigating three CCA cell lines established from OV-related CCA patients (HuCCA-1, KKU-100, and KKU-M055) using immunoblotting revealed that all CCA cells consistently expressed high levels of PDGFR-α and low levels of PDGFR-β. Among them, KKU-100 exhibited the highest expression of PDGFR-α, while PDGFR-β was equally expressed in all cells (Figure 1C).

**Figure 2:**
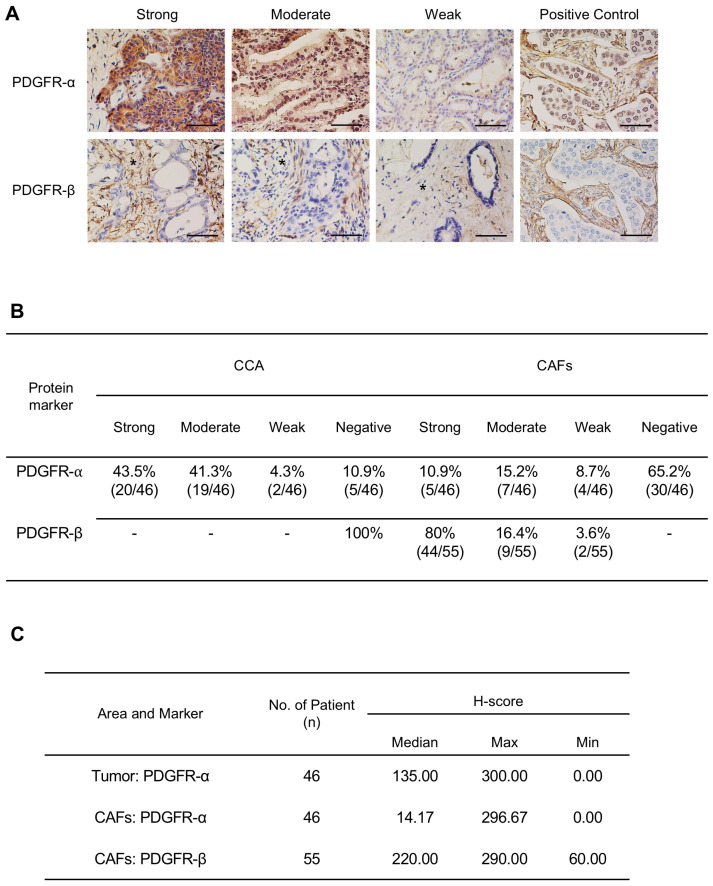
(**A**) Representative images showing the expression level of PDGFR-α and PDGFR-β in Thai-CCA tissues. The expression intensity was evaluated separately in CCA tumor cells and cancer-associated fibroblasts (CAFs) (3+, strong staining; 2+, moderate staining; and 1+, weak staining). Breast cancer tissues were used as a positive control for PDGFR. Asterisks indicate stromal area. Scale bars = 50 μm. (**B**) Percentage of CCA tissues expressing PDGFR-α and PDGFR-β in CCA tumors and CAFs. (**C**) H-score values of PDGFR-α and PDGFR-β expression in tumor and CAF areas.

Immunohistochemistry analysis demonstrated positive staining for PDGFR-α in all CCA cases, with most presenting moderate or strong staining in CCA cells, and 34.8% of cases (16/46) exhibited varied intensities of PDGFR-α in the cytoplasmic area of CAFs. In contrast, PDGFR-β displayed moderate to strong staining in CAFs in 96.4% of cases (53/55) and was negatively stained in CCA cells (Figure 2A,B). Semi-quantitative assessment using H-score analysis revealed a median H-score of 135 for PDGFR-α in tumors and 14.17 for CAFs. For PDGFR-β in CAFs, the median H-score was 220 (Figure 2C). The expression level of PDGFR was graded as low (weak intensity, H-score < 70) or high (moderate to strong intensity, H-score ≥ 70). We explored the association between high PDGFR-α and PDGFR-β expression and clinicopathological parameters. The results indicated a significant association of high expressions of PDGFR-α in CCA cells and PDGFR-β in CAFs with the stage and survival rates (Table S2 and Figure S1 in Supplementary Materials). CCA patients with high PDGFR-α expression in tumor cells had a median survival time of 25 months, compared to 51.5 months for those with low expression (*p* = 0.0471). Similarly, patients with high PDGFR-β expression in CAFs had median survival times of 17.5 months, while those with moderate expression had a median survival time of 40 months (*p* = 0.0198) (Figure S1 in Supplementary Materials). These findings strongly support the involvement of PDGFR-α and PDGFR-β in OV-related CCA.

### 2.2. Sensitivity of PDGFR Inhibitors

**Figure 3:**
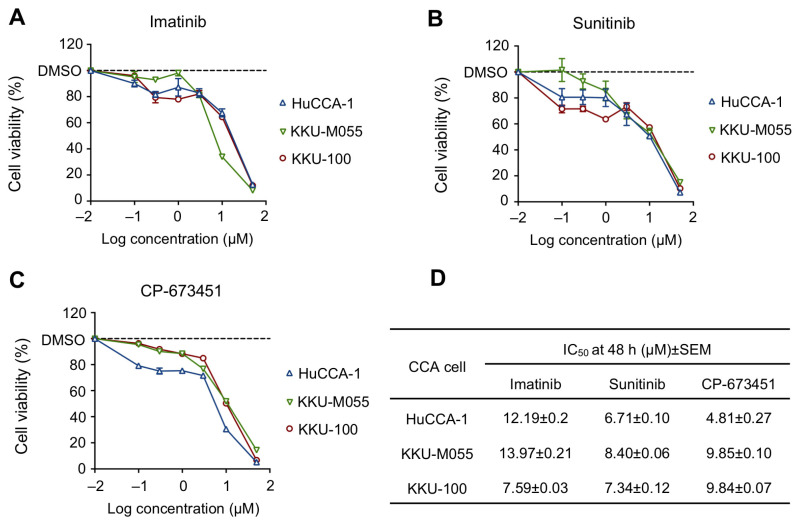
Cell viability of CCA cell lines treated with (**A**) Imatinib, (**B**) Sunitinib, and (**C**) CP-673451 for 48 h at the indicated concentrations, evaluated by MTT assay. (**D**) IC_50_ of PDGFR inhibitors in CCA cell lines. Data are shown as a mean ± SEM of triplet individual experiments.

The sensitivity of PDGFR inhibitors, including imatinib, sunitinib, and CP-673451, was investigated for their toxicity in CCA cells using the MTT assay. The result demonstrated that all PDGFR inhibitors exhibited a dose-dependent decrease in CCA cells (Figure 3A–C), with half maximal inhibitory concentration (IC_50_) values ranging from 4.81 to 13.97 μM (Figure 3D). Specifically, HuCCA-1 cells showed the highest sensitivity to CP-673451 (IC_50_ = 4.81 μM), while KKU-M055 and KKU-100 showed the highest sensitivity to sunitinib (IC_50_ = 8.40 μM and 13.97 μM, respectively). Notably, there was no apparent correlation between the protein expression levels of PDGFR-α and PDGFR-β and the response to the PDGFR inhibitors (IC_50_) (Table S3 in Supplementary Materials). We selected the drug with the lowest IC_50_, CP-673451, and the most sensitive cells, HuCCA-1, for further investigation.

### 2.3. CP-673451 Induced Apoptosis in HuCCA-1 Cells

**Figure 4:**
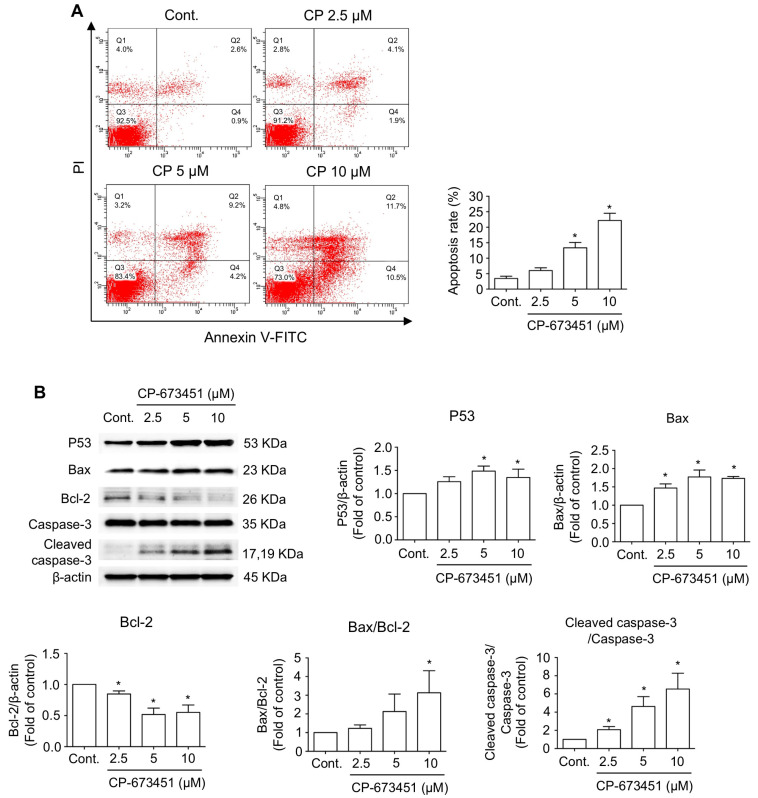
CP-673451-induced CCA cell apoptosis. (**A**) HuCCA-1 cells were incubated with CP-673451 for 24 h at the indicated concentrations, and apoptosis was examined by flow cytometry using Annexin V-FITC and PI staining. (**B**) Apoptosis proteins including P53, Bax, Bcl-2, caspase-3, and cleaved caspase-3 were examined in HuCCA-1 cell lysates after CP-673451 treatment for 12 h by Western blotting. Β-actin was used as an internal control. Data are shown as a mean ± SEM of triplet individual experiments. * specifies a significant difference from the control group at *p* < 0.05.

To examine the effect of CP-673451 on cell death in HuCCA-1 cells, apoptosis was evaluated. Flow cytometry analysis of Annexin V-FITC/Propidium iodide (Pl) staining demonstrated that CP-673451 treatment dose-dependently induced both early-stage apoptosis (Annexin V+/PI−, represented in Quadrant 4 (Q4)) and late-stage apoptosis (Annexin V+/PI+, represented in Q2) in CCA cells, with a significant effect found at concentrations of 5 and 10 µM (Figure 4A). Furthermore, Western blot analysis revealed an increased level of pro-apoptotic proteins, including P53, Bax, and cleaved caspase-3, along with a decreased level of the anti-apoptotic protein Bcl-2 (Figure 4B).

### 2.4. CP-673451 Induced ROS Elevation

**Figure 5:**
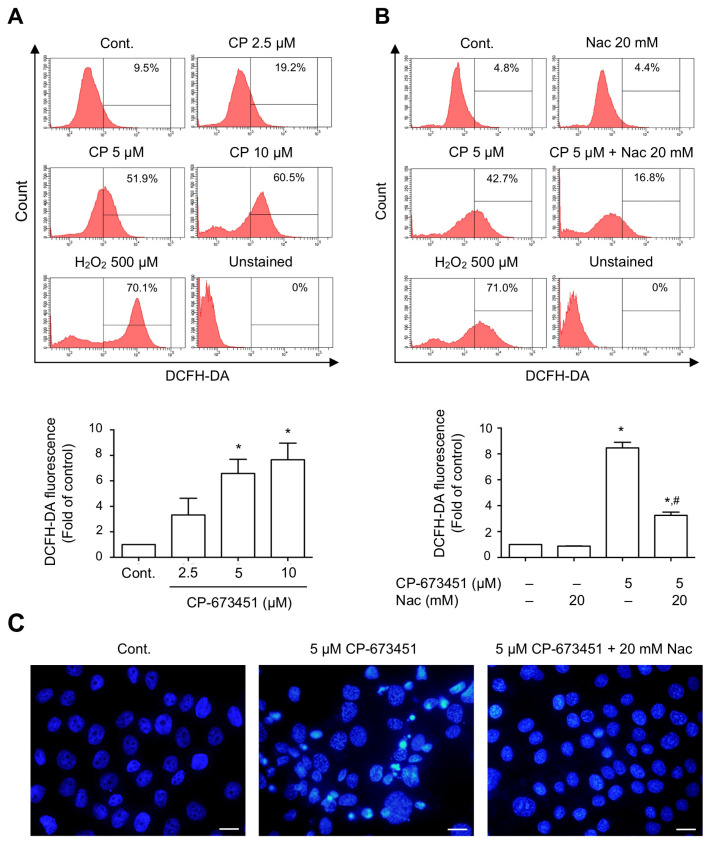
CP-673451-induced ROS elevation in CCA cells. Flow cytometry analysis demonstrating (**A**) 5 and 10 μM CP-673451 significantly induced increased cellular ROS level, while (**B**) administration of Nac (20 mM) efficiently reversed the effect of CP-673451 on HuCCA-1 cells. (**C**) Fluorescent micrographs showing DAPI-stained apoptotic nuclei of CCA cells treated with CP-673451. Treatment of CCA cells with Nac could protect cells from CP-673451-induced apoptosis. Scale bars = 20 μm. Data are shown as a mean ± SEM of triplet individual experiments. * and # indicate significant difference from control and CP-673451 group, respectively, at *p* < 0.05.

We further investigated whether apoptosis resulted from increased ROS levels induced by CP-673451. The DCFH-DA fluorescence assay showed that CP-673451 dose-dependently induced the production of ROS in HuCCA-1 cells (Figure 5A). To validate whether CP-673451 induced apoptosis via ROS elevation, we added N-acetylcysteine (Nac), a ROS-scavenging substrate, along with CP-673451 treatment. The result showed that the administration of Nac reversed the effect of CP-673451-promoted ROS elevation and apoptosis in HuCCA-1 cells (Figure 5B,C). These findings strongly support the notion that inhibiting PDGFR-α with CP-673451 leads to ROS elevation, resulting in the induction of apoptosis in CCA cells.

### 2.5. CP-673451 Suppressed Nrf2 Protein Expression and Nuclear Translocation and Inhibited PI3K/Akt Pathway

**Figure 6:**
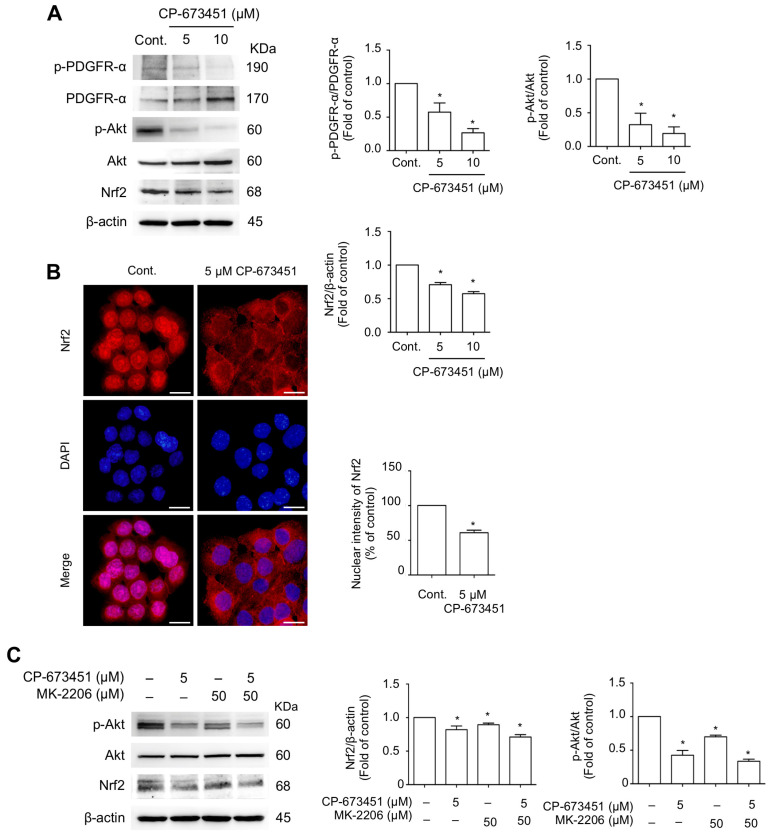
CP-673451 inhibited PDGFR-mediated PI3K/Akt/Nrf2 signaling. (**A**) Western blot analysis of p-PDGFR-α, PDGFR-α, p-Akt, Akt, and Nrf2 in HuCCA-1 cells treated with CP-673451 for 6 h at the indicated concentrations. (**B**) Immunofluorescent images showing Nrf2 nuclear translocation (red) in CCA cells. Cell nuclei were stained with DAPI (blue). HuCCA-1 cells were incubated with 5 μM CP-673451 for 3 h, probed with an anti-Nrf2 antibody, and photographed under a fluorescent microscope. Bar graphs represent the averaged nuclear intensity of Nrf2 determined in six random fields. Scale bars = 20 μm. (**C**) Western blot analysis of p-Akt, Akt, and Nrf2 in HuCCA-1 cells treated with CP-673451 (5 µM), MK-2206 (50 µM), and MK-2206 combined with CP-673451. Β-actin was used as an internal control. Data are shown as a mean ± SEM of triplet individual experiments. * indicates a significant difference from the control group at *p* < 0.05.

Cellular ROS levels are regulated by the redox-sensitive transcription factor, nuclear factor erythroid 2-related factor 2 (Nrf2) [24]. The PI3K/Akt pathway, a major signaling pathway of PDGFR-α activation [25], acts as an upstream regulator of Nrf2 [26]. We examined the effect of CP-673451 on the downstream signaling pathway of PDGFR-α and Nrf2 in HuCCA-1 cells. The results indicated that HuCCA-1 cells constitutively exhibited a high activity of PDGFR-α and Akt, as shown by elevated levels of phosphorylated PDGFR-α and phosphorylated Akt in the control cells. Treatment with CP-673451 for 6 h effectively decreased the levels of p-PDGFR/PDGFR, p-Akt/Akt, and Nrf2 (Figure 6A). Furthermore, immunofluorescent staining showed that CP-673451 caused a decreased nuclear translocation of Nrf2 compared to the control (Figure 6B).

Next, we compared the effect of CP-673451 on the decreased level of Akt with a highly selective Akt inhibitor, MK-2206, and determined the level of Nrf2 expression. The results revealed that MK-2206 single treatment had an inhibitory effect on p-Akt, which was related to decreased Nrf2 expression, and this effect was similar to the CP-673451 treatment (Figure 6C). Additionally, when the cells were treated concurrently with MK-2206 and CP-673451, no additive reduction effect on p-Akt and Nrf2 levels was observed. These results strongly suggest that CP-673451 mediates the inhibition of Nrf2 expression through the PDGFR/PI3K-Akt pathway.

### 2.6. CP-673451 Decreased the Expression of Antioxidant Genes in HuCCA-1 Cells

**Figure 7:**
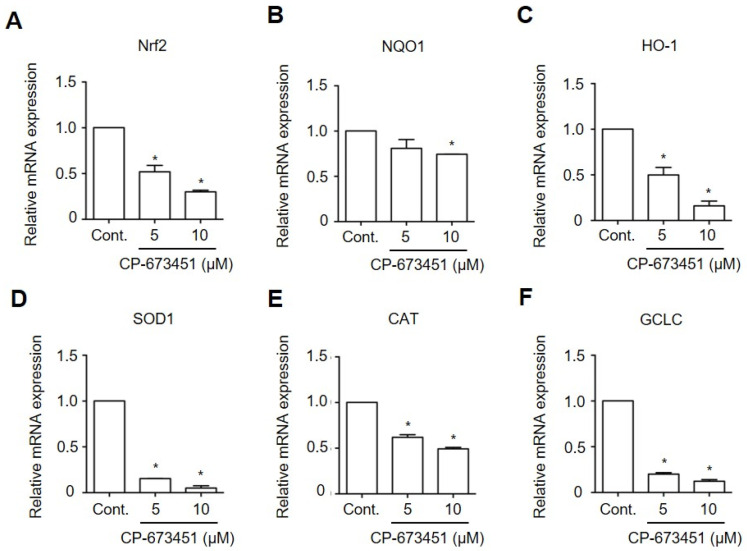
CP-673451 reduced the expression of Nrf2 and their target genes in CCA cells. HuCCA-1 cells were incubated with CP-673451 at the indicated concentrations for 6 h. mRNA expressions of (**A**) Nrf2 and their target genes, including (**B**) NQO1, (**C**) HO-1, (**D**) SOD1, (**E**) CAT, and (**F**) GCLC, were analyzed by RT-qPCR. Data are shown as a mean ± SEM of triplet individual experiments. NQO1, NAD(P)H:quinone oxidoreductase 1; HO-1 Heme oxygenase-1; SOD1, Superoxide dismutase; CAT, Catalase; GCLC, Glutamate–Cysteine Ligase Catalytic Subunit. * indicates a significant difference from the control group at *p* < 0.05.

Nrf2 plays a crucial role in controlling the expression of phase II antioxidant enzymes, including NAD(P)H: quinone oxidoreductase 1 (NQO1), Heme oxygenase-1 (HO-1), Superoxide dismutase 1 (SOD1), Catalase (CAT), and Glutamate–Cysteine Ligase Catalytic Subunit (GCLC), each acting in oxidative stress elimination [24]. To investigate the effect of CP-673451 on Nrf2 activity, we conducted RT-qPCR to examine the expression of these phase II antioxidant genes. The results demonstrated that the exposure of HuCCA-1 cells to CP-673451 for 6 h led to a dose-dependent decrease in the expression of Nrf2 and all the phase II antioxidant enzymes mRNAs (Figure 7).

## 3. Discussion

Cholangiocarcinoma (CCA) is a highly desmoplastic neoplasm characterized by the presence of cancer-associated fibroblasts (CAFs) in the tumor microenvironment [27]. The interplay between tumor cells and CAFs is regulated by platelet-derived growth factor (PDGF)-PDGF receptor (PDGFR) signaling [15]. In the present study, we examined the expression levels of PDGF and PDGFR in *Opisthorchis viverrini* (OV)-related CCA tissues and various Thai CCA cell lines. We provide evidence that a highly selective PDGFR-targeted drug, CP-673451, is a promising therapeutic drug. Furthermore, we demonstrate insights into the mechanisms of CP-673451-induced apoptosis in CCA cells.

Our study of 58 cases of intrahepatic cholangiocarcinoma (ICC) revealed an elevated mRNA expression of all PDGF isoforms (PDGF-A, PDGF-B, PDGF-C, and PDGF-D), along with their corresponding receptors PDGFR-α and PDGFR-β in cancerous tissues compared to adjacent hepatocyte-rich tissue. Immunohistochemistry analysis for PDGFR-α and PDGFR-β exhibited negative staining in hepatocytes and normal bile ducts in normal liver tissue [28,29]. PDGFR-α was expressed in both CCA cells and the surrounding stromal CAFs, while PDGFR-β was predominantly expressed in CAFs [15]. Notably, our findings linked the high expression of PDGFR-α and PDGFR-β in tumor tissues with advanced tumor stages and shorter survival times. Additionally, the expression of PDGFR-β correlated with PDGF-B expression. Collectively, these observations suggest a distinctive role for PDGF isoforms and PDGFR in the autocrine/paracrine pathway between CAFs and CCA cells, contributing to the pathogenesis of OV-related CCA. Previous studies have reported increased expression levels of PDGFR-α and PDGF-A in OV-induced CCA, suggesting their potential involvement in OV-related CCA carcinogenesis [18]. Consequently, PDGFR-α and PDGF-A represent promising targets for therapeutic intervention in CCA. Further exploration of their roles may provide valuable insights into potential treatment strategies for CCA.

PDGFR inhibitors, such as imatinib and sunitinib, have been evaluated for the treatment of CCA in both preclinical and clinical trials. In a phase II clinical trial for advanced CCA, imatinib demonstrated limited activity [30]. On the other hand, sunitinib has shown promise due to its activity against various tyrosine kinases involved in tumor growth and angiogenesis [31]. While sunitinib is currently undergoing phase II clinical studies as a second-line treatment for advanced-stage CCA [11], its overall efficacy in CCA treatment remains to be firmly established. CP-673451, a selective inhibitor of the PDGFR tyrosine kinase, shows potential as a therapeutic strategy. However, its efficacy in OV-related CCA cells has yet to be examined, representing a research gap that necessitates further exploration.

In our study, we examined the sensitivity of PDGFR inhibitors, specifically imatinib, sunitinib, and CP-673451, in OV-associated CCA cell lines—HuCCA-1, KKU-M055, and KKU-100. Investigating the basal expression levels of PDGFR-α and PDGFR-β, we found that KKU-100 exhibited the highest expression of PDGFR-α, while all three CCA cell lines showed only a slight expression of PDGFR-β. However, the level of PDGFR-α expression did not demonstrate a significant correlation with drug sensitivity. This finding is consistent with previous research, which reported no definitive link between PDGFR expression and responsiveness to PDGFR inhibitors [32]. These data suggest that, in our tested CCA cell lines, high PDGFR expression may not reliably predict treatment response [33]. Genetic differences, such as mutations of PDGFR, KRAS, and TP53, may contribute to variations in drug responses among different CCA cell lines [14,34,35,36]. Our findings are consistent with a study on OV-related CCA cells and epidermal growth factor receptor (EGFR) kinase inhibitors, suggesting that drug sensitivity does not consistently correlate with EGFR expression levels [37]. In non-small cell lung cancer (NSCLC), patients with low or moderate EGFR expression have shown better responses to EGFR inhibitors than those with high EGFR expression, emphasizing the role of EGFR mutation status in predicting drug sensitivity [38,39]. These observations emphasize the complexity of predicting drug responsiveness solely based on receptor expression levels and highlight the need for a more sophisticated understanding of the genetic landscape in CCA. Further investigations into the molecular mechanisms influencing drug sensitivity will be crucial for tailoring effective therapeutic strategies for OV-related CCA.

In OV-related CCA tissues, Boonjaraspinyo et al. (2012) [20] identified point mutations in PDGFR-α, including silent and missense mutations. Silent mutations were observed in the tyrosine kinase domains, while missense mutations occurred in the dimerization domain. Interestingly, no activating mutations of PDGFR-α were found. This study also highlighted significant expressions of PDGFR-α and PDGF-A, indicating the presence of an autocrine/paracrine stimulation loop in CCA. These findings suggest that treating CCA with tyrosine kinase inhibitors could be effective [20]. However, there is no information available about the mutation status of PDGFR-β in CCA.

It has been observed that CCA patients with a positive KRAS mutation tend to have worse outcomes compared to those without the mutation [40]. Furthermore, TP53 mutations have been shown to reduce the sensitivity to drugs targeting tyrosine kinases [36]. The low sensitivity of CP-673451 in KKU-100 and KKU-M055 cells can be attributed to the presence of KRAS and TP53 mutations in KKU-100 [41] and the MEK mutation in KKU-M055 [35]. On the other hand, HuCCA-1 cells had the highest sensitivity (lowest IC_50_) to the specific PDGFR inhibitor CP-673451. Unfortunately, there are no data available on the mutation status of HuCCA-1 cells. A recent study by Jamnongsong et al. (2022) on drug response profiling in OV-related CCA cells classified HuCCA-1, KKU-100, and KKU-M055 into different drug response subgroups, each showing distinct responses to inhibitors through various signaling pathways [42]. Notably, PDGFR inhibitors were not included in their drug response analyses.

The PI3K-Akt signaling pathway serves as the main intracellular signal transduction pathway of PDGFR [25]. A previous study has reported that the multitarget inhibitors, including imatinib and sunitinib, suppress CCA activities by decreasing the expression of p-PDGFR-α and p-AKT [20]. PI3K-Akt signaling is the crucial upstream regulator of Nrf2 [26]. When activated, Nrf2 acts as a transcription factor by translocating into the nucleus and activating the expression of antioxidant genes [24]. The constitutive activation of Nrf2 in cancer cells facilitates cancer growth and resistance to therapeutic drugs [43]. The present study provides evidence that CP-673451 disrupts the PI3K/Akt signaling pathway, reduces Nrf2 expression and nuclear translocation, and thereby decreases the expression of antioxidant genes. This reduction in antioxidant enzymes results in elevated cellular ROS levels and upregulated P53 expression, subsequently increasing the ratio of Bax/Bcl-2 [44], resulting in an increased cleaved caspase-3 expression, a key executor of cell apoptosis [45].

We investigated whether decreased cell viability by CP-673451 also resulted from reduced cell proliferation by employing a colony-forming assay and Western blotting for proliferation markers. We observed a slight decrease in the number of CCA colonies, and there were no significant changes in the protein expression levels of the proliferation markers P21, cyclin D1, and cyclin E. These findings suggest that the reduced cell viability caused by CP-673451 may be due to the induction of CCA cell apoptosis rather than the suppression of CCA cell proliferation.

Our study has demonstrated the importance of PDGFR signaling for the survival and progression of CCA cells. The findings also highlight the potential of CP-673451, a drug targeting PDGFR, which could be an effective treatment for CCA. However, it is important to note that high PDGFR expression alone cannot reliably predict drug sensitivity, due to genetic variations such as PDGFR mutations. This makes it challenging to understand the essential survival pathways associated with CP-673451-resistant CCA. Previous studies have shown that tyrosine kinase signaling pathways are significantly involved in the development of CCA, and tyrosine kinase inhibitors have emerged as promising candidates for CCA treatments [8,9,10,37]. Further research should explore the combined effects of CP-673451 with other tyrosine kinase inhibitors, such as EGFR, FGFR, and VEGFR inhibitors, or chemotherapeutic agents to enhance the effectiveness of CCA treatments. These studies can help us develop more comprehensive and efficacious strategies in the management of CCA.

## 4. Materials and Methods

### 4.1. Cell Lines and CCA Tissue Samples

Human CCA cells related to fluke infection, including HuCCA-1, KKU-M055, and KKU-100, were used in this study. All cell lines were obtained from the Japanese collection of research bioresources (JCRB) Cell bank (Osaka, Japan). HuCCA-1 (JCRB1657), KKU-M055 (JCRB1551), and KKU-100 (JCRB1568) were cultured in RPMI medium supplemented with 10% fetal bovine serum (Gibco Invitrogen, Carlsbad, CA, USA), 1.13 g/L sodium bicarbonate, and 1% antibiotic–antimycotic (Gibco Invitrogen, Carlsbad, CA, USA) at 37 °C in a 5% CO_2_ atmosphere.

A total of fifty-eight intrahepatic CCA tissues used in this study included archived samples and surgical resection samples collected from the Department of Pathology and Department of Surgery, Ramathibodi Hospital, Mahidol University, Thailand, with the approval of the ethics committee, Faculty of Medicine Ramathibodi Hospital, Mahidol University (protocol no. 12-58-41). Hematoxylin and eosin (H&E) staining was performed in 5 µm thick CCA tissue sections for histological analysis. The histological identification of CCA tissue was performed by the two medical pathologists (NL and CS). The tissue samples were categorized as the mass-forming cholangiocarcinoma subtype.

Thai patients were diagnosed as OV-associated CCA if at least one out of these five parameters were fulfilled: (1) positive stool examination for OV or their eggs in the previous record; (2) stool or bile contains OV or their eggs; (3) a typical bead-like cholangiogram identification; (4) illustration of small peripheral intrahepatic bile duct dilation, accordant with parasitic diseases of the biliary tract on sonography, magnetic resonance image (MRI), or computed tomography (CT); and (5) the hepatic resected sample contained histopathological evidence of OV. The representative areas of cancer epithelium cells and adjacent stroma were identified, and tissue cores were constructed for the tissue microarray (TMA) as previously described [46].

### 4.2. Immunohistochemistry

Fifty-eight intrahepatic CCA tissues were subject to immunohistochemical analysis using a Leica Bond-Max automation (Leica, Bannockburn, IL, USA). After deparaffinization, hydration, and antigen retrieval, the TMA slides were incubated with the anti-PDGFR-α and anti-PDGFR-β primary antibodies (Santa Cruz Biotechnology, Santa Cruz, CA, USA), followed by biotinylated secondary antibody, and detected using DAB chromogenic agent. Hematoxylin was used for slide counterstaining. Breast cancer tissue was used as the positive control, and the section stained with an isotype-matched control antibody without the primary antibody was used as the negative control. The immunohistochemical data were evaluated by two pathologists blinded to the clinical information. The staining of tumor cells and cancer-associated fibroblasts (CAFs) was scored separately using the H-score method [47], which was analyzed by a semi-quantitative evaluation of both the intensity of staining (graded as 0 for non-staining; 1 for weak; 2 for moderate; or 3 for strong) and the percentage of positive cells. The scores ranged from 0 to 300. The H-score was calculated as follows:

H-score = (% cells with weak staining × 1) + (% cells with moderate staining × 2) + (% cells with strong staining × 3)

The H-score value of expression was defined as follows: <35 denotes no expression, 36–69 denotes weak expression, 70–149 denotes moderate expression, and ≥150 denotes strong expression.

### 4.3. Analysis of RNA Expression

Total RNA was extracted from sixteen pairs of intrahepatic CCA and matched resection margins, and from three CCA cell lines using Tri-reagent (Molecular Research Center, Inc., Cincinnati, OH, USA). The Revert Aid first-strand cDNA synthesis kit (Thermo Scientific, Waltham, MA, USA) was used based on the manufacturer’s protocols for reverse transcription. The expression of mRNA transcripts was quantified by reverse transcription-quantitative polymerase chain reaction (RT-qPCR) using the specific primer sequences listed in Table 1. A PCR reaction mixture was prepared in a final volume of 10 μL consisting of 1 μL of cDNA, 3 μL of nuclease-free water, 0.5 μL of 10 μM forward and reverse primers, and 5 μL SYBR green master mix (Bio-Rad Laboratories, Hercules, CA, USA). PCR was performed in a Bio-Rad CFX96 Real-Time PCR System (Bio-Rad Laboratories, Hercules, CA, USA) under the following conditions: 3 min initial denaturation at 95 °C, 40 amplification cycles of denaturation (5 s at 95 °C), annealing (30 s at 59 °C), and extension (30 s at 72 °C). The threshold cycle (Ct) of each gene was measured and normalized against GAPDH mRNA expression. The relative mRNA expression level was calculated using the software Bio-Rad CFX Manager version 3.1 (Bio-Rad Laboratories, Hercules, CA, USA) based on the 2^−∆∆Ct^ method.

### 4.4. Drug-Sensitivity Assay In Vitro

CCA cells were grown at a density of 5 × 10^3^ cells/well on 96-well plates and then incubated with PDGFR-targeted drugs including imatinib (targeted Abl, PDGFR, Kit), sunitinib (targeted PDGFR, VEGFR, Kit, Flt3), and CP-673451 (targeted PDGFR) (Selleckchem, Houston, TX, USA) for 48 h. Viable cell percentage was examined using the methylthiazolium bromide (MTT) assay. After inhibitor treatment, the cells were incubated with MTT solution (100 μL, 500 μg/mL) (Sigma Aldrich, St. Louis, MO, USA) at 37 °C in the dark for 3 h, and then the formazan crystals were solubilized by adding dimethyl sulfoxide (DMSO) solution (100 μL) (Hi-Media, Mumbai, Maharashtra, India). The absorbance was measured using a Versamax microplate reader using SoftMax^®^ Pro 4.8 analysis software (Molecular Devices, Union City, CA, USA) at 490 nm.

### 4.5. Western Blot Analysis

This study investigated the basal expression of PDGFR-α and PDGFR-β in CCA cells and the expression of PDGFR downstream signaling proteins after CP-673451 treatment. Our preliminary results demonstrated that CP-673451 showed a dose- and time-dependent decrease in p-PDGFR level in CCA cells, and the significant decrease was found at 6 h after treatment at concentrations of 5 and 10 µM. HuCCA-1 cells were grown overnight at a density of 5 × 10^5^ cells/well on 6-well plates before drug treatment. Cells were treated with different concentrations of CP-673451 (2.5, 5, and 10 μM) for 12 h for the determination of proteins involved in apoptosis and at concentrations of 5 and 10 μM for 6 h for the determination of PDGFR downstream signaling proteins. Cells were also simultaneously treated with a potent Akt inhibitor, 50 μM of MK-2206 (Selleckchem, Houston, TX, USA), to validate whether the Akt pathway is involved in CP-673451-induced CCA cell death. The total protein lysate was extracted from CCA cells using the mammalian protein extraction reagent, M-PER (ThermoFisher, Pittsburgh, PA, USA), with a protease inhibitor cocktail (Santa Cruz Biotechnology, CA, USA). Protein concentration was determined by BCA assay using the Pierce BCA Protein Assay Kit (Thermo Scientific, Waltham, MA, USA). The protein sample (25 μg) was separated on SDS-PAGE (8% gel for PDGFR and downstream signaling proteins and 15% gel for apoptosis-associated proteins) and transferred onto a nitrocellulose membrane (Merck, Darmstadt, Germany). After blocking with 3% bovine serum albumin (Capricorn Scientific, Ebsdorfergrund, Germany) for 1.5 h, the membranes were incubated at 4 °C overnight with the following primary antibodies: anti-PDGFR-α, anti-PDGFR-β, anti-Bax, anti-Bcl-2, anti-caspase 3, anti-phospho-Akt 1/2/3, anti-β-actin (Santa Cruz, CA, USA), anti-P53, anti-cleaved caspase 3, anti-phospho-PDGFR-α (pTyr849) (Cell Signaling Technology Inc., Beverly, MA, USA), and anti-Nrf2 (Abcam, Cambridge, UK). After washing with PBS, membranes were incubated with the secondary antibodies for 2 h at room temperature including horseradish peroxidase-conjugated anti-rabbit or anti-mouse IgG (Cell Signaling Technology Inc., Beverly, MA, USA). The visualization of protein expression was performed using enhanced chemiluminescence (ECL) (Bio-Rad Laboratories, Hercules, CA, USA) and detected by an Alliance Q9 mini chemiluminescent imaging system (UVITEC, Cambridge, UK). The protein band intensities were normalized with β-actin using ImageJ 1.47 software (from the NIH website by Scion Corporation, Frederick, MD, USA) and expressed as the fold of control.

### 4.6. Apoptosis Analysis

#### 4.6.1. DAPI Staining

HuCCA-1 cells were grown on glass coverslips in 24-well plates at a density of 1.5 × 10^5^ cells/well. Then, they were incubated for 24 h with CP-673451 with or without N-acetylcysteine (Nac) (Temmler Pharma, Marburg, Germany). After fixing the cells with 4% formaldehyde for 10 min, the cells were stained with DAPI for 5 min. After washing twice with PBS, cell nuclei were imaged using an Olympus BX53 upright fluorescence microscope (Olympus, Tokyo, Japan).

#### 4.6.2. Flow-Cytometry

HuCCA-1 cells were grown at a density of 5 × 10^5^ cells/well on 6-well plates and then incubated with CP-673451 for 24 h. After incubation, cells were harvested and stained with FITC Annexin-V/Dead Cell Apoptosis Kit (V13242; Invitrogen, Grand Island, NY, USA) following the manufacturer’s protocols. Briefly, the harvested cells were washed with cold PBS, resuspended in Annexin-binding buffer, mixed gently with Annexin V (5 μL) and 100 μg/mL PI (1 μL), and incubated for 15 min in the dark. After resuspending the stained cells in Annexin-binding buffer, the apoptotic cells were analyzed using a BD FACSCanto™ flow cytometer (BD Bioscience, San Jose, CA, USA), in which 3 × 10^4^ cells were counted per sample. The BD FACSDIVA 6.1.3 software (BD Biosciences, CA, USA) was performed for data analysis.

### 4.7. Intracellular Reactive Oxidative Species (ROS) Level

HuCCA-1 cells at a density of 5 × 10^5^ cells/well were grown on 6-well plates and then incubated with CP-673451 and/or Nac for 12 h. After incubation, cells were harvested and incubated with 10 μM of DCFH-DA (2′,7′-dichlorofluorescein diacetate) (Sigma, St. Louis, MO, USA) for 25 min at 37 °C in the dark. The stained cells were suspended in PBS and analyzed using a flow cytometer, in which 3 × 10^4^ cells were counted per sample.

### 4.8. Immunofluorescence

HuCCA-1 cells were grown on glass coverslips in 24-well plates at a density of 1.5 × 10^5^ cells/well in 24-well plates and then incubated with CP-673451 for 3 h. Cells were fixed for 10 min with 4% formaldehyde, permeabilized for 10 min with 0.1% Triton X-100, and blocked for 1 h with 5% normal goat serum and 3% BSA. Then, the cells were incubated overnight with an anti-Nrf2 primary antibody (Abcam, Cambridge, UK), followed by 1 h incubation in the dark with an Alexa Fluor 594 conjugated goat anti-mouse secondary antibody (Invitrogen, Carlsbad, CA, USA). DAPI was performed for nuclei staining. Cells were visualized with an Olympus BX53 upright fluorescence microscope (Olympus, Tokyo, Japan), and the intensity of Nrf2 immunostaining was determined using ImageJ software (from the NIH website by Scion Corporation, Frederick, MD, USA).

### 4.9. Statistical Analysis

All experiments were performed in triplicate. The results are represented as means ± the standard error of the mean (SEM) and statistically analyzed using GraphPad 6 Software (GraphPad Software, Inc., San Diego, CA, USA). Student’s unpaired *t*-test (two-tailed) was performed for the comparison between the two groups. One-way ANOVA followed by Tukey’s multiple comparison tests was applied for comparing more than two experimental groups. A statistically significant difference was considered when the *p*-value was less than 0.05. The associations between PDGFRs or PDGF ligand expression and clinicopathological parameters, PDGFR inhibitor sensitivity (IC_50_), and PDGFR-α or PDGFR-β protein expression were analyzed using Pearson’s correlation coefficient.

## 5. Conclusions

In conclusion, this study reports a high expression of PDGF and PDGFR in OV-related CCA tissues and demonstrates the selective inhibition against PDGFR, CP-673451, as a potential treatment for OV-related CCA patients. This study also elucidates the underlying mechanism of PDGFR-drug-induced apoptosis in CCA cells. However, this study lacks data on the drug’s effect on stromal cells such as CAFs, which also express PDGFR at high levels and play a role in conjunction with CCA cells in tumor progression. Therefore, further exploration of the drug’s response in CAFs and in vivo studies is essential for a better understanding of the drug’s effects and its potential clinical implications in the future.

## Figures and Tables

**Table 1:** Primer sequences for qRT-PCR.

Gene	Primer Sequences (5′-3′)	Amplicon Size (bp)	Accession Number
PDGFR-α	F: TTGAAGGCAGGCACATTTACA R: GCGACAAGGTATAATGGCAGAAT	119	NM_006206.5
PDGFR-β	F: CCTGCAATGTGACGGAGAGT R: GGTGCGGTTGTCTTTGAACC	200	NM_002609.3
PDGF-A	F: GCAAGACCAGGACGGTCATTT R: GGCACTTGACACTGCTCGT	135	NM_033023.4
PDGF-B	F: CACTCGATCCGCTCCTTTGA R: CGGGTCATGTTCAGGTCCAA	92	NM_033016.3
PDGF-C	F: GCCAGGTTGTCTCCTGGTTA R: TGCTTGGGACACATTGACAT	86	NM_016205.3
PDGF-D	F: GTGGAGGAAATTGTGGCTGT R: CGTTCATGGTGATCCAACTG	172	NM_033135.3
NRF2	F: AGGTTGCCCACATTCCCAAA R: AGTGACTGAAACGTAGCCGA	118	NM_006164.5
NQO1	F: GTTTGGAGTCCCTGCCATTC R: AAGCACTGCCTTCTTACTCCG	118	NM_000903.3
HO-1	F: AACTTTCAGAAGGGCCAGGT R: CTGGGCTCTCCTTGTTGC	112	NM_002133.3
SOD1	F: ACAAAGATGGTGTGGCCGAT R: AACGACTTCCAGCGTTTCCT	162	NM_000454.5
CAT	F: GCCACAGGAAAGTACCCCTC R: GAGGCCAAACCTTGGTGAGA	106	NM_001752.4
GCLC	F: GAGGTCAAACCCAACCCAGT R: AAGGTACTGAAGCGAGGGTG	92	NM_001498.4
GAPDH	F: CTGACTTCAACAGCGACACC R: TGCTGTAGCCAAATTCGTTG	114	NM_002046.7

## Data Availability

The data presented in this study are available on request from the corresponding author.

## Supplementary Materials

The following supporting information can be downloaded at https://www.mdpi.com/article/10.3390/ph17010009/s1. Figure S1: Kaplan–Meier survival analysis of CCA patients according to IHC level of PDGFR-α expression in tumor and PDGFR-β expression in CAFs, analyzed by Log-rank (Mantel-Cox) test; Table S1: Relationship between PDGFR and PDGF mRNA expression; Table S2: Correlation of high PDGFR expression and clinicopathological parameters; Table S3: Relationship between the PDGFR protein expression and IC_50_ of PDGFR inhibitors.

## References

[1] IARC (1994). Infection with liver flukes (Opisthorchis viverrivi, Opisthorchis felineus and Clonorchis sinensis). IARC Monogr. Eval. Carcinog. Risks Hum..

[2] Sripa B., Brindley P.J., Mulvenna J., Laha T., Smout M., Mairiang E., Bethony J.M., Loukas A. (2012). The tumorigenic liver fluke Opisthorchis viverrini-multiple pathways to cancer. Trends Parasitol..

[3] Montori M., Scorzoni C., Argenziano M.E., Balducci D., De Blasio F., Martini F., Buono T., Benedetti A., Marzioni M., Maroni L. (2022). Cancer-associated fibroblasts in cholangiocarcinoma: Current knowledge and possible implications for therapy. J. Clin. Med..

[4] Khan S.A., Davidson B.R., Goldin R., Pereira S.P., Rosenberg W.M.C., Taylor-Robinson S.D., Thillainayagam A.V., Thomas H.C., Thursz M.R., Wasan H. (2002). Guidelines for the diagnosis and treatment of cholangiocarcinoma: Consensus document. Gut.

[5] Bridgewater J.A., Palmer D., Cunningham D., Iveson T., Gillmore R., Waters J., Harrison M., Valle J.W., Wasan H., Corrie P. (2012). Second-line therapy in advanced biliary tract cancer: Baseline data from a retrospective multi-centre series. Ann. Oncol..

[6] Sia D., Hoshida Y., Villanueva A., Roayaie S., Ferrer J., Tabak B., Peix J., Sole M., Tovar V., Alsinet C. (2013). Integrative molecular analysis of intrahepatic cholangiocarcinoma reveals 2 classes that have different outcomes. Gastroenterology.

[7] Yoshikawa D., Ojima H., Iwasaki M., Hiraoka N., Kosuge T., Kasai S., Hirohashi S., Shibata T. (2008). Clinicopathological and prognostic significance of EGFR, VEGF, and HER2 expression in cholangiocarcinoma. Br. J. Cancer.

[8] Lee P., Hendifar A., Osipov A., Cho M., Li D., Gong J. (2021). Targeting the fibroblast growth factor receptor (FGFR) in advanced cholangiocarcinoma: Clinical trial progress and future considerations. Cancers.

[9] Jin W. (2020). ErBb family proteins in cholangiocarcinoma and clinical implications. J. Clin. Med..

[10] Smyth E.C., Sclafani F., Cunningham D. (2014). Emerging molecular targets in oncology: Clinical potential of MET/hepatocyte growth-factor inhibitors. OncoTargets Ther..

[11] Neuzillet C., Seitz J.-F., Fartoux L., Malka D., Lledo G., Tijeras-Raballand A., Gramont A.D., Ronot M., Bouattour M., Dreyer C. (2015). Sunitinib as second-line treatment in patients with advanced intrahepatic cholangiocarcinoma (SUN-CK phase II trial): Safety, efficacy, and updated translational results. J. Clin. Oncol..

[12] Reinmuth N., Liersch R., Raedel M., Fehrmann F., Fehrmann N., Bayer M., Schwoeppe C., Kessler T., Berdel W., Thomas M. (2009). Combined anti-PDGFRα and PDGFRβ targeting in non-small cell lung cancer. Int. J. Cancer.

[13] Ko Y.J., Small E.J., Kabbinavar F., Chachoua A., Taneja S., Reese D., DePaoli A., Hannah A., Balk S.P., Bubley G.J. (2001). A multi-institutional phase ii study of SU101, a platelet-derived growth factor receptor inhibitor, for patients with hor-mone-refractory prostate cancer. Clin. Cancer Res..

[14] Heinrich M.C., Corless C.L., Demetri G.D., Blanke C.D., von Mehren M., Joensuu H., McGreevey L.S., Chen C.J., Van den Abbeele A.D., Druker B.J. (2003). Kinase mutations and imatinib response in patients with metastatic gastrointestinal stromal tumor. J. Clin. Oncol..

[15] Cadamuro M., Nardo G., Indraccolo S., Dall’olmo L., Sambado L., Moserle L., Franceschet I., Colledan M., Massani M., Stecca T. (2013). Platelet-derived growth factor-D and Rho GTPases regulate recruitment of cancer-associated fibroblasts in cholangiocarcinoma. Hepatology.

[16] Huang F., Wang D., Yao Y., Wang M. (2017). PDGF signaling in cancer progression. Int. J. Clin. Exp. Med..

[17] Demoulin J.B., Essaghir A. (2014). PDGF receptor signaling networks in normal and cancer cells. Cytokine Growth Factor Rev..

[18] Boonjaraspinyo S., Wu Z., Boonmars T., Kaewkes S., Loilome W., Sithithaworn P., Nagano I., Takahashi Y., Yongvanit P., Bhudhisawasdi V. (2012). Overexpression of PDGFA and its receptor during carcinogenesis of Opisthorchis viverrini-associated chol-angiocarcinoma. Parasitol. Int..

[19] Omenetti A., Popov Y., Jung Y., Choi S.S., Witek R.P., Yang L., Brown K.D., Schuppan D., Diehl A.M. (2008). The hedgehog pathway regulates remodelling responses to biliary obstruction in rats. Gut.

[20] Boonjaraspinyo S., Boonmars T., Wu Z., Loilome W., Sithithaworn P., Nagano I., Pinlaor S., Yongvanit P., Nielsen P.S., Pairojkul C. (2012). Platelet-derived growth factor may be a potential diagnostic and prognostic marker for cholangiocarcinoma. Tumour Biol..

[21] Fingas C.D., Mertens J.C., Razumilava N., Bronk S.F., Sirica A.E., Gores G.J. (2012). Targeting PDGFR-β in cholangiocarcinoma. Liver Int..

[22] Roberts W.G., Whalen P.M., Soderstrom E., Moraski G., Lyssikatos J.P., Wang H.F., Cooper B., Baker D.A., Savage D., Dalvie D. (2005). Antiangiogenic and antitumor activity of a selective PDGFR tyrosine kinase inhibitor, CP-673,451. Cancer Res..

[23] Xi Y., Chen M., Liu X., Lu Z., Ding Y., Li D. (2014). CP-673451, a platelet-derived growth-factor receptor inhibitor, suppresses lung cancer cell proliferation and migration. OncoTargets Ther..

[24] Nguyen T., Sherratt P.J., Pickett C.B. (2003). Regulatory mechanisms controlling gene expression mediated by the antioxidant re-sponse element. Annu. Rev. Pharmacol. Toxicol..

[25] Wang H., Yin Y., Li W., Zhao X., Yu Y., Zhu J., Qin Z., Wang Q., Wang K., Lu W. (2012). Over-expression of PDGFR-β promotes PDGF-induced proliferation, migration, and angiogenesis of EPCs through PI3K/Akt signaling pathway. PLoS ONE.

[26] Jeong W.S., Jun M., Kong A.N. (2006). Nrf2: A potential molecular target for cancer chemoprevention by natural compounds. Antioxid. Redox Signal..

[27] Biffi G., Tuveson D.A. (2021). Diversity and biology of cancer-associated fibroblasts. Physiol. Rev..

[28] Hayes B.J., Riehle K.J., Shimizu-Albergine M., Bauer R.L., Hudkins K.L., Johansson F., Yeh M.M., Mahoney W.M., Yeung R.S., Campbell J.S. (2014). Activation of platelet-derived growth factor receptor alpha contributes to liver fibrosis. PLoS ONE.

[29] Kocabayoglu P., Lade A., Lee Y.A., Dragomir A.C., Sun X., Fiel M.I., Thung S., Aloman C., Soriano P., Hoshida Y. (2015). β-PDGF receptor expressed by hepatic stellate cells regulates fibrosis in murine liver injury, but not carcinogenesis. J. Hepatol..

[30] Wiedmann M.W., Mossner J. (2010). Molecular targeted therapy of biliary tract cancer-results of the first clinical studies. Curr. Drug Targets.

[31] Mendel D.B., Laird A.D., Xin X., Louie S.G., Christensen J.G., Li G., Schreck R.E., Abrams T.J., Ngai T.J., Lee L.B. (2003). In vivo antitumor activity of SU11248, a novel tyrosine kinase inhibitor targeting vascular endothelial growth factor and plate-let-derived growth factor receptors: Determination of a pharmacokinetic/pharmacodynamic relationship. Clin. Cancer Res..

[32] Basciani S., De Luca G., Dolci S., Brama M., Arizzi M., Mariani S., Rosano G., Spera G., Gnessi L. (2008). Platelet-derived growth factor receptor β-subtype regulates proliferation and migration of gonocytes. Endocrinology.

[33] Froehner M., Beuthien-Baumann B., Dittert D.D., Schuler U., Wirth M.P. (2006). Lack of efficacy of imatinib in a patient with metastatic Leydig cell tumor. Cancer Chemother. Pharmacol..

[34] Hirota S., Ohashi A., Nishida T., Isozaki K., Kinoshita K., Shinomura Y., Kitamura Y. (2003). Gain-of-function mutations of platelet-derived growth factor receptor α gene in gastrointestinal stromal tumors. Gastroenterology.

[35] Lau D.K., Mouradov D., Wasenang W., Luk I.Y., Scott C.M., Williams D.S., Yeung Y.H., Limpaiboon T., Iatropoulos G.F., Jenkins L.J. (2019). Genomic profiling of biliary tract cancer cell lines reveals molecular subtypes and actionable drug targets. iScience.

[36] Canale M., Petracci E., Delmonte A., Chiadini E., Dazzi C., Papi M., Capelli L., Casanova C., De Luigi N., Mariotti M. (2017). Impact of TP53 mutations on outcome in EGFR-mutated patients treated with first-line tyrosine kinase inhibitors. Clin. Cancer Res..

[37] Boonsri B., Yacqub-Usman K., Thintharua P., Myint K.Z., Sae-Lao T., Collier P., Suriyonplengsaeng C., Larbcharoensub N., Balasubramanian B., Venkatraman S. (2021). Effect of combining EGFR tyrosine kinase inhibitors and cytotoxic agents on cholangiocarcinoma cells. Cancer Res. Treat..

[38] Shepherd F.A., Rodrigues Pereira J., Ciuleanu T., Tan E.H., Hirsh V., Thongprasert S., Campos D., Maoleekoonpiroj S., Smylie M., Martins R. (2005). Erlotinib in previously treated non-small-cell lung cancer. N. Engl. J. Med..

[39] Fukuoka M., Yano S., Giaccone G., Tamura T., Nakagawa K., Douillard J.Y., Nishiwaki Y., Vansteenkiste J., Kudoh S., Rischin D. (2003). Multi-institutional randomized phase II trial of gefitinib for previously treated patients with advanced non-small-cell lung cancer (The IDEAL 1 Trial). J. Clin. Oncol..

[40] Yokoyama M., Ohnishi H., Ohtsuka K., Matsushima S., Ohkura Y., Furuse J., Watanabe T., Mori T., Sugiyama M. (2016). KRAS mutation as a potential prognostic biomarker of biliary tract cancers. Jpn. Clin. Med..

[41] Saensa-Ard S., Leuangwattanawanit S., Senggunprai L., Namwat N., Kongpetch S., Chamgramol Y., Loilome W., Khansaard W., Jusakul A., Prawan A. (2017). Establishment of cholangiocarcinoma cell lines from patients in the endemic area of liver fluke infection in Thailand. Tumor Biol..

[42] Jamnongsong S., Kueanjinda P., Buraphat P., Sakornsakolpat P., Vaeteewoottacharn K., Okada S., Jirawatnotai S., Sam-pattavanich S. (2022). Comprehensive drug response profiling and pan-omic analysis identified therapeutic candidates and prognostic biomarkers for Asian cholangiocarcinoma. iScience.

[43] Mitsuishi Y., Motohashi H., Yamamoto M. (2012). The Keap1-Nrf2 system in cancers: Stress response and anabolic metabolism. Front. Oncol..

[44] Cheng E.H., Wei M.C., Weiler S., Flavell R.A., Mak T.W., Lindsten T., Korsmeyer S.J. (2001). Bcl-2, Bcl-X(L) sequester BH3 do-main-only molecules preventing Bax and Bak-mediated mitochondrial apoptosis. Mol. Cell.

[45] Cheng Y.X., Liu R., Wang Q., Li B.S., Xu X.X., Hu M., Chen L., Fu Q., Pu D.M., Hong L. (2012). Realgar-induced apoptosis of cervical cancer cell line Siha via cytochrome c release and caspase-3 and caspase-9 activation. Chin. J. Integr. Med..

[46] Kononen J., Bubendorf L., Kallionimeni A., Bärlund M., Schraml P., Leighton S., Torhorst J., Mihatsch M.J., Sauter G., Kallioniemi O.P. (1998). Tissue microarrays for high-throughput molecular profiling of tumor specimens. Nat. Med..

[47] Mazières J., Brugger W., Cappuzzo F., Middel P., Frosch A., Bara I., Klingelschmitt G., Klughammer B. (2013). Evaluation of EGFR protein expression by immunohistochemistry using H-score and the magnification rule: Re-analysis of the SATURN study. Lung Cancer.

