# Supplementary material for: CP-673451, a Selective Platelet-Derived Growth Factor Receptor Tyrosine Kinase Inhibitor, Induces Apoptosis in Opisthorchis viverrini-Associated Cholangiocarcinoma via Nrf2 Suppression and Enhanced ROS
Source: Pharmaceuticals (Basel). 2023 Dec 20;17(1):9. doi: 10.3390/ph17010009 (PMC10821224; doi:10.3390/ph17010009)
Supplement: Supplementary file 1 [file pharmaceuticals-17-00009-s001.zip › pharmaceuticals-2754229-supplementary.pdf]

S1 Table. Correlation of PDGFR and PDGF mRNA expression

Gene	*p*-value				
	PDGFR-α	PDGFR-β	PDGF-A	PDGF-B	PDGF-C
PDGFR-β	<0.0001	-	-	-	-
PDGF-A	0.1707	0.8668	-	-	-
PDGF-B	0.0013	0.0119	0.0216	-	-
PDGF-C	0.7437	0.662	0.0272	0.4896	-
PDGF-D	0.046	0.0018	0.9191	0.0457	0.5617

Positive correlation

S2 Table. Correlation of high PDGFR expression and clinicopathological parameters

Location and Marker	Parameter	No. of Patient	*P*-value
Tumor:	Tumor stages		
High PDGFR-α expression	I & II	10	0.0142
	III & IV	9	
	Tumor size		
	Size < 5 cm	6	0.0030
	Size ≥ 5 cm	20	
CAFs:	Tumor stages		
High PDGFR-β expression	I & II	10	0.0251
	III & IV	11	

S3 Table. Relationship between the PDGFR protein expression and IC_{50} of PDGFR inhibitors.

Expression		IC_{50} of PDGFR inhibitors		
		Imatinib	Sunitinib	CP-673451
PDGFR-α	*p*-value	0.9196	0.5657	0.1719
PDGFR-β	*p*-value	0.5008	0.1194	0.3478

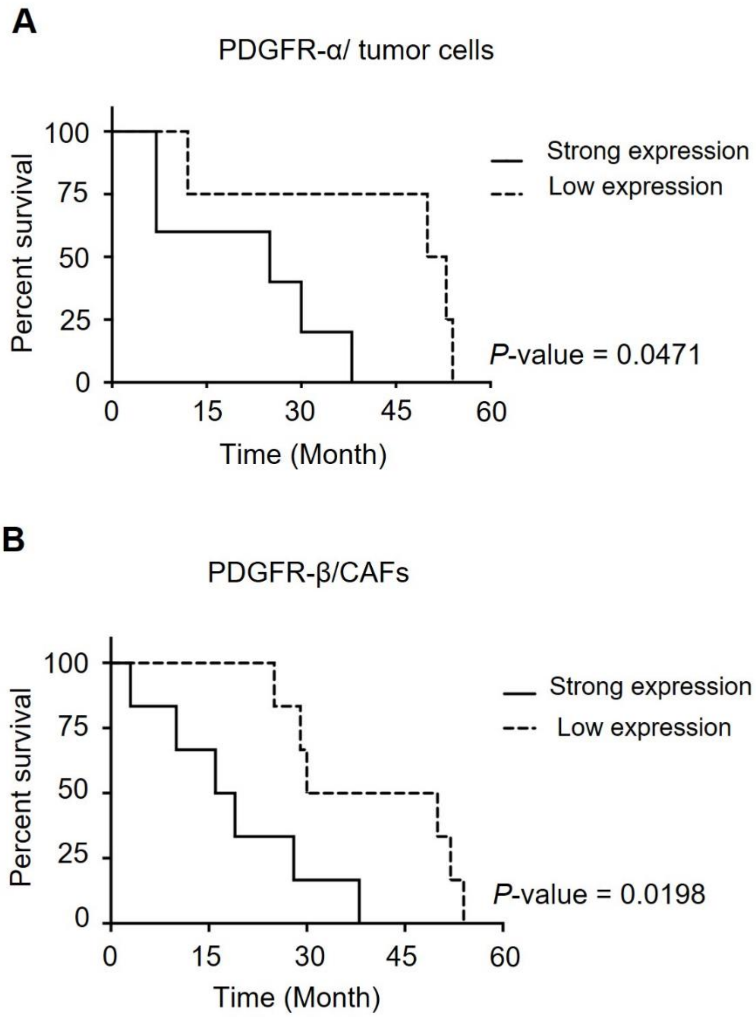

S1 Fig. Kaplan-Meier survival analysis of CCA patients according to IHC level of (A) PDGFR-α expression in tumor and (B) PDGFR-β expression in CAFs, analyzed by Log-rank (Mantel-Cox) test.
